# The Science of the Future: Establishing a Citizen-Scientist Collaborative Agenda After Covid-19

**DOI:** 10.3389/fpubh.2020.00282

**Published:** 2020-06-05

**Authors:** Livio Provenzi, Serena Barello

**Affiliations:** ^1^Child Neurology and Psychiatry Unit, IRCCS Mondino Foundation, Pavia, Italy; ^2^EngageMinds HUB, Consumer, Food & Health Engagement Research Center, Department of Psychology, Università Cattolica del Sacro Cuore, Milan, Italy

**Keywords:** COVID-19 (2019-nCoV), coronavirus, science, ethics, citizen science, public health, media

While we are writing, many countries are still dealing with Covid-19 outbreak and many are moving toward a gradual easing of the restrictive measures. In the meantime, the role of scientists in the public community is silently, yet dramatically changing. Decision-makers are asking research experts to provide evidence on which political acts should be grounded. Citizens are insecure and they wonder how they are supposed to protect themselves from the contagion and when the vaccines will be available (1). The media coverage of the pandemic features a daily presence of scientists and public health professionals on the television and on the web, highlighting the key role of experts to deal with the emergency, but making also clear that any solution is still far from being conclusive.

It is increasingly becoming evident that the time needed for public health and scientific advance exceeds the time expected by citizens to obtain satisfactory responses. In a way, science is slow, uncertain, discordant, and fragile; and the increasing public awareness of its probabilistic nature (2, 3) may change the public perception of scientific knowledge for a long time. Lessons learned from previous epidemics suggest that disasters can affect the public understanding of science and the citizens' trust in scientist and experts (4). As for the Covid-19 emergency, a recent Italian study by Battiston et al. (5) suggests that while an initial increase in attention and information-seeking from scientists was registered on social media in February—the very start of the epidemic in Italy—, a dramatic decline in trust toward scientific and health authorities occurred in March 2020. As a result, the unprecedented and massive exposure of science to the public—together with the lack of definitive responses to citizens' needs—risks to end in a dramatic loss of trust in science (5).

## The Citizen Scientists and the COVID-19 Emergency

Of course, science represents the best product of human cultural evolution. And still it is the kind of knowledge we can rely on to cope with this unprecedented worldwide healthcare emergency (4). Notwithstanding, the scientific field is now at an historical turning-point that should not be underestimated. Researchers have now the opportunity to redefine their relationships with the society. The notion of the “citizen scientist” has been increasingly highlighted in many different contexts in which the reciprocal partnership and engagement among researchers, citizens and policy makers was recognized as key to the success of multi-stakeholders initiatives (6). In environmental health, citizen science informs research questions, data collection and analysis, and conclusions that can impact the quality of life in local environments. The active citizens' participation in large-scale genome projects can empower even marginalized groups and minorities in shaping scientific inquiry through participation (7). The distributed availability of smart devices catalyze the potentials of citizen-driven data for many different scientific fields, from public health to biology, from physics to ecology.

Now, the Covid-19 emergency is requiring that science and society work together to share needs, resources, actions and solutions. For example, in order to develop accurate and reliable models of the contagion spread, researchers need citizens to allow their real-time position tracking through apps and devices, thus making them the frontline data collector subjects (8). At the same time, clinicians and researchers need citizens to be engaged in respecting the mitigation and containment norms to adequately deal and reduce the virus spread (9). Citizens also need to trust scientists and researchers, as they are the experts holding theoretical and pragmatical knowledge, skills and resources to achieve the discovery of reliable and effective pharmacological therapies for the Covid-19. Honestly, this is not new. But the community impact of Covid-19 and its media coverage is increasing the awareness of citizens about the role of research for their life (10). Moreover, previous research suggests that the heightened media exposure to Ebola-related stories was related to increased distress, worries, and confusion in the citizens (11).

## Public Health Opportunities for Scientific Citizenship

While this pandemic is challenging the relationship between science and the people, it is also true—as it is for any crisis in life—that it holds potentials and opportunities. Here, we would like to highlight some of the valuable opportunities hidden in the actual pandemic for the evolution of public health science. In general, these potentials require us to consider the active engagement of citizens as a pivotal—rather than ancillary or secondary—element in every research step (12).

First, the communication of science to naïve readers and the public is challenged. Although many scientists may still be reluctant in investing time and resources in public communication of science and technology (13), reach-out communications cannot be anymore supplementary. Rather they need to be a relevant element of scientific plans. Not surprisingly, during the last decade, reach-out communication strategies are increasingly requested by international funding agencies as core elements of research projects and they contribute to the evaluation process of scientific applications. As the Covid-19 health emergency is paralleled by the risk of a pandemic of social media panic (14), these communications should favor citizens' comprehension and curiosity, rather than serve sensationalistic goals that may ultimately increase panic reactions.

Second, science should communicate and explain its processes in a way that is robust, yet understandable by the public. Public communication of science during the Covid-19 emergency should promote—more than ever—the exchange of balanced information and the engagement of the citizens as necessary active participants in a complex health information environment (15). How are findings obtained? What does it mean that a finding is “true” or “reliable” from a probabilistic point of view? Scientists should be able to explain—avoiding technical jargon—why research findings are something to be understood, and not something that require faith.

Third, truly collaborative models will need to be guided and shaped by flexible yet clear guidelines (16). Although there might be skepticism among some researchers about the quality of citizen-collected data (7), both methodological and statistical approaches are now available to promote high-quality citizen science projects. Collaborative models of science should reside in the middle of the spectrum between citizen- and scientist-initiated projects (17). The development, validation and acceptance of these guidelines (16) should be itself a co-designed initiative in which both researchers and citizens should play an active and dialogic role. The opportunity to invest in participatory citizen science projects during and after the Covid-19 pandemic should not be loss. Specific directions for advancing the field include (a) improving secure open-source data management tools, (b) promoting projects with real and concrete local effects for citizens in the place where they live, (c) and creating and/or strengthening networks of research consortiums to reduce redundancy and optimize resources among different local projects (7).

Fourth, without a proper methodological education, the “citizen scientist” may easily become an empty claim among scientists (12); a value with which no one of us disagree, but that ultimately fail in promoting reciprocal and mutually beneficial partnerships. To avoid this risk, scientists should be educated to recognize that citizens are already engaged in science by definition, as everything in science talks about them (16). Delivering effective education programs is a core part of the citizen science agenda (7). Investing in the education of new generations of scientists and researchers on citizen science appears to be a major goal of science educational programs after the Covid-19 outbreak in order to value and advance citizens' agency in science.

## Paving the way for the Public Health Science of the Future

The Covid-19 is probably going to change our lives for many years to come. The socio-economic and emotional burden of this pandemic will require relevant efforts from government and social community and the societies that will re-emerge from this 2020 emergency will no longer be the same. So, there is no reason to believe that science itself—and the way it produces narratives about its progress—will not be affected. More than this, we argue that public health research should be transformed, in order to take its role in the responsible steering of the post-Covid-19 society to a new form of participatory and collaborative engagement approach to research. The partnership among citizens, clinicians and scientists is no longer deferrable and the year 2020 appears to be a point of no return to plan the science of the future.

## Author Contributions

SB and LP conceived the ideas behind this work and refined the final version for submission. LP drafted the initial version of the manuscript. Both authors agreed on final submission.

## Conflict of Interest

The authors declare that the research was conducted in the absence of any commercial or financial relationships that could be construed as a potential conflict of interest.
